# New findings on CD16^bright^CD62L^dim^ neutrophil subtypes in sepsis-associated ARDS: an observational clinical study

**DOI:** 10.3389/fimmu.2024.1331050

**Published:** 2024-03-28

**Authors:** Jing Zhang, Chencheng Gao, Zhenxing Zhu, Danyang Li, Lai Qu, Qiuli Xue, Guoqiang Wang, Tong Ji, Fang Wang

**Affiliations:** ^1^ Department of Pathogeny Biology, College of Basic Medical Sciences, Jilin University, Changchun, China; ^2^ Department of Critical Care Medicine, The First Hospital of Jilin University, Changchun, China; ^3^ Department of Hematology and Oncology, The Third Hospital of Jilin University, Changchun, China

**Keywords:** acute respiratory distress syndrome, CD16 bright CD62L dim neutrophil subtype, IL-8, sepsis, immunosuppression

## Abstract

**Background:**

The CD16^bright^CD62L^dim^ neutrophil subtype is a recently identified neutrophil subtype. The aim of this study was to evaluate changes of peripheral blood CD16^bright^CD62L^dim^ neutrophils in patients with sepsis-associated ARDS.

**Methods:**

We prospectively recruited adult patients with sepsis-associated ARDS in the intensive care unit (ICU). Patient demographic data, medical history information, and laboratory data were collected within 48 hours of enrollment, and flow cytometry was applied to analyze the CD16^bright^CD62L^dim^ neutrophil subtype in the patients’ peripheral blood. Multifactor COX regression models were used to analyze factors affecting prognosis, and Spearman correlation coefficients were used to analyze clinical and laboratory indicators affecting complications of infection.

**Results:**

Of the 40 patients, 9 patients died by the 28-day follow-up, indicating a mortality rate of 22.5%. Patients in the nonsurvival group had higher CD16^bright^CD62L^dim^ neutrophil levels. Patients with sepsis-associated ARDS who had a baseline proportion of CD16^bright^CD62L^dim^ neutrophil subtypes to total neutrophils in peripheral blood >3.73% had significantly higher 28-day mortality, while patients with CD16^bright^CD62L^dim^ neutrophil subtypes counts >2.62×10^9^/L were also associated with significantly higher 28-day mortality. The percentage of the CD16^bright^CD62L^dim^ neutrophil subtype (HR=5.305, 95% CI 1.986-14.165, p=0.001) and IL-8 (HR=3.852, 95% CI 1.561-9.508, p=0.003) were independent risk factors for the development of infectious complications in patients with sepsis-related ARDS. The percentage of CD16^bright^CD62L^dim^ neutrophil subtypes predicted an AUC of 0.806 (95% CI 0.147-0.964, P=0.003) for the development of infectious complications, and 0.742 (95% CI 0.589-0.895, P=0.029) for the prediction of death within 28 days.

**Conclusion:**

We identified for the first time that CD16^bright^CD62L^dim^ neutrophils are elevated in patients with sepsis-associated ARDS and are associated with infectious complications and poor prognosis. The percentage of CD16^bright^CD62L^dim^ neutrophil subtypes may serve as a predictor of the development of infectious complications in patients with ARDS.

## Introduction

Acute respiratory distress syndrome (ARDS) is a common critical disease among patients admitted to the intensive care unit (ICU), with a prevalence of approximately 10.4% among ICU patients (1). ARDS is a secondary condition that occurs within 7 days (most commonly within 6-48 hours) following the onset of a primary illness of multifactorial etiology (most commonly pneumonia and extrapulmonary sepsis) (2) and is most often associated with a severe systemic inflammatory response. Over the past four decades, with advances in care and improved treatment strategies for ARDS, early mortality in the clinical course of patients has decreased, and the overall survival of patients with ARDS is improving (3, 4). Unfortunately, mortality in ARDS patients remains as high as 35% (1), and there has been a dramatic increase in the number of ARDS patients transitioning to persistent inflammation, immunosuppression and catabolism syndrome (PICS); associated complications include prolonged stays in the ICU, high resource utilization, increased discharges to nonhome sites, sepsis recurrence requiring readmission, persistent cognitive and functional impairment, and low long-term survival (5).

Sepsis is a life-threatening organ dysfunction caused by a dysregulated host response to infection and is a common clinical critical illness that is often associated with multiorgan failure, immune dysregulation, and high mortality (6). Sepsis is a major risk factor in ARDS and is responsible for approximately 75% of ARDS cases (1). ARDS can lead to a substantial increase in the morbidity and mortality of septic patients (7, 8). Studies have shown that immune disorders in patients with sepsis are an important cause of their poor prognosis (9).

The CD16^bright^CD62L^dim^ neutrophil subtype is a recently identified neutrophil subtype characterized by immunosuppressive functions (10–12). Clinical studies have confirmed that this subtype is significantly elevated in the peripheral blood of patients after severe trauma and may be associated with reduced immune function in trauma patients and with increased secondary opportunistic infections (13). A similarly elevated proportion of heterogeneous neutrophil subtypes has been found in the peripheral blood of ARDS patients (14), but whether the elevated proportion represents the CD16^bright^CD62L^dim^ neutrophil subtype is unknown; furthermore, information on its relevance to immunosuppression and prognosis in ARDS patients is limited.

In this study, we aimed to assess changes in peripheral blood CD16^bright^CD62L^dim^ neutrophil subtypes in patients with sepsis-associated ARDS and to evaluate factors associated with infectious complications and poor prognosis in patients with sepsis-related ARDS.

## Methods

### Study design and patients

This was a prospective observational study of adult sepsis-related ARDS patients admitted to our ICU between January 2021 and August 2021. Inclusion criteria were as follows: 1) patients ≥18 and ≤90 years of age admitted to the ICU who met criteria for sepsis (6) (detail in **Supplementary Materials**); 2) patients with sepsis receiving mechanical ventilation (MV) for hypoxic respiratory failure, whether invasive mechanical ventilation (IMV) or noninvasive mechanical ventilation (NIMV), while meeting diagnostic criteria for ARDS (3) (detail in **Supplementary Materials**). The exclusion criteria were as follows: 1) near-death status or death within 48 hours of enrollment; 2) pregnant patients and patients in the puerperium; 3) patients with immune abnormalities (15) (details in **Supplementary Materials**); and 4) patients with a prolonged stay in medical care centers (unable to be discharged after more than one month).

### Data collection and evaluation

Demographic data and medical history information were collected within 24 hours of patient admission, including age, sex, underlying disease, reason for ICU admission, sepsis of the infected site, whether vasoactive drugs were applied, Acute Physiology and Chronic Health Evaluation (APACHE) II score, Sequential Organ Failure Assessment (SOFA) score, and Glasgow Coma Scale (GCS) score. Determination of sepsis, septic shock and ARDS required consensus among 2 or more clinicians not involved in this study and evaluation of diagnostic procedures, therapeutic procedures, respiratory support and other clinical events, such as infectious complications.

Data representing laboratory parameters were collected within 48 hours of patient enrollment, including routine blood count, arterial blood gas measurements, procalcitonin (PCT), fungal (1-3)-β-D glucan, C-reactive protein (CRP), peripheral blood inflammatory factor levels, and lymphocyte subpopulation counts.

An ICU physician not involved in this study resolved inconsistencies in the investigator-entered data based on a comparison of study case report forms with medical charts.

### Microbiological evaluation and diagnostic criteria

According to the treatment routine, we collected sputum, blood and urine at the time of patient enrollment and twice a week during treatment. Pleural fluid, peritoneal fluid, and bronchoalveolar lavage fluid were also collected when available, and these samples were subjected to pathogenic culture. Imaging of possible infection sites was completed regularly (once a week or when the condition changed) according to the advice of the treating physician and depending on the patient’s condition.

### Neutrophil isolation and flow cytometry assay

Since the CD16^bright^CD62L^dim^ neutrophil subtype is present at extremely low levels in patients, to successfully identify this neutrophil subtype, we collected 4 ml fresh anticoagulated blood samples from enrolled patients, applied the Human Peripheral Blood Neutrophil Isolation Kit (Stemcell, Canada), and isolated approximately 5×10^6^ neutrophils from each specimen. Neutrophils were stained with flow antibodies, including anti-CD16-FITC (eBioscience, USA) and anti-CD62L-APC (eBioscience, USA), at room temperature and protected from light for 30 min. Finally, neutrophils were analyzed by multicolor flow cytometry (ACEABIO, USA), and the results were processed by FlowJo v10.0.7 software (Tree Star, Ashland, OR, USA).

### Clinical outcomes

The primary outcome was 28-day mortality, and other outcomes were the appearance of infectious complications (details in **Supplementary Materials**).

### Statistical analysis

All data were processed by applying SPSS23 software. The measurement data were tested for normality, nonnormally distributed measurement data were described using the median (interquartile spacing), and the Mann−Whitney test was used for comparisons between groups. The count data were described using frequency (percentage), and the chi-square test was used for comparison between groups. The cutoff values were calculated using jamovi for each laboratory index grouping, the survival curves under different index groupings were plotted using Kaplan−Meier, the differences in survival curves were compared using the log-rank test. A multifactor binary logistic stepwise regression analysis was applied to include variables with p < 0.1 in the univariate analysis, the receiver operating characteristic curve (ROC) was plotted, and the area under the curve (AUC) was calculated, and the prognostic influences were analyzed using a multifactor COX regression model. Correlation analysis of clinical and laboratory indicators was performed using the Spearman correlation coefficient. Differences were considered statistically significant at P < 0.05.

## Results

### Study population and prevalence of ARDS

**Figure 1:**
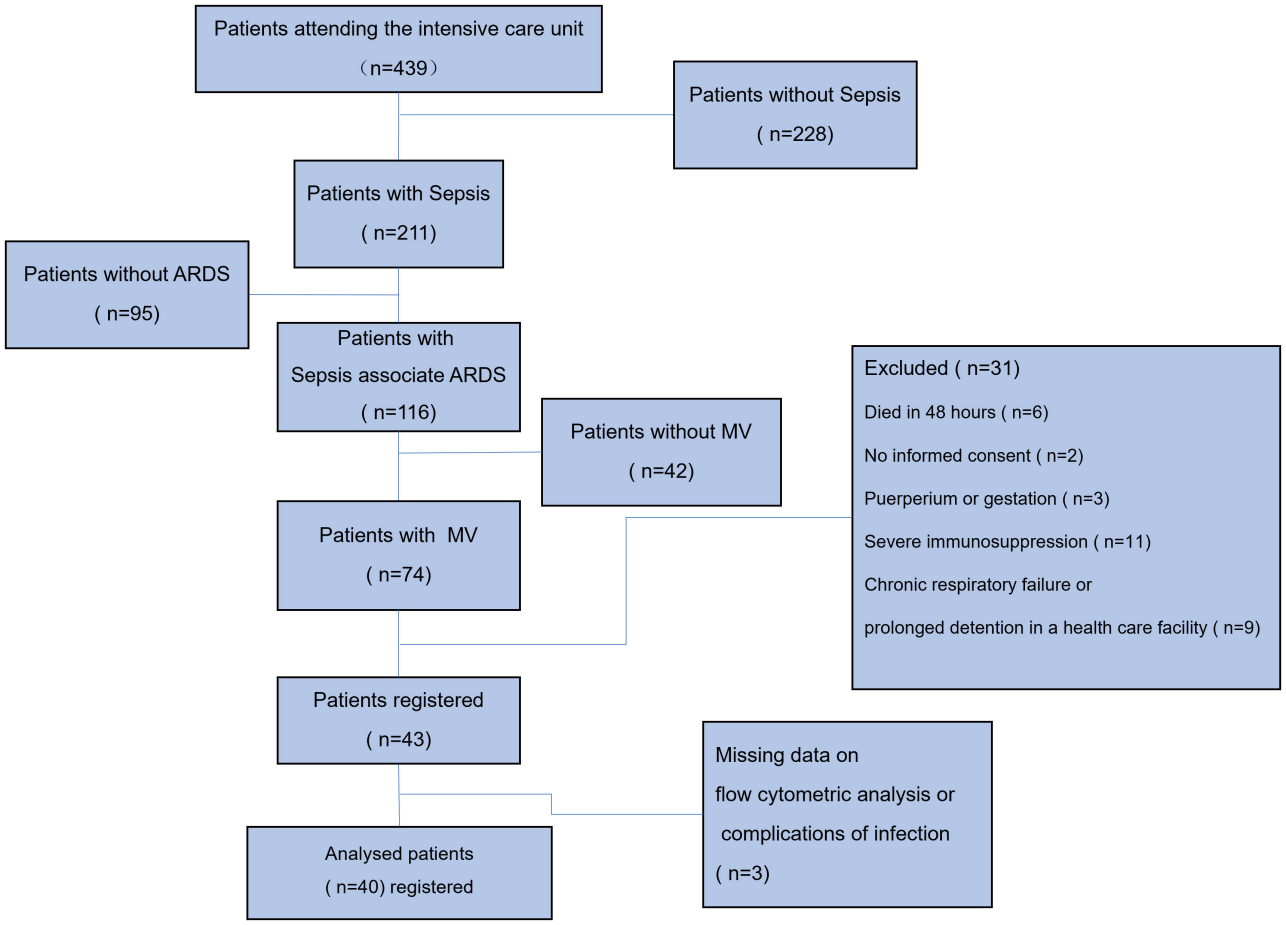
Study flow chart. A total of 439 ICU patients were investigated during the study period, of whom 211 (48.06%) met the diagnostic criteria for sepsis or septic shock while in the ICU. Hypoxic respiratory failure was present in 116 (54.98%) of these patients according to the Berlin ARDS criteria, but 42 (36.21%) of them were not mechanically ventilated, and 74 (63.69%) were mechanically ventilated (both NIMV and IMV). Of these 74 patients, 6 died within 48 hours of ICU admission, 2 patients failed to provide informed consent, 3 were pregnant or in the puerperium, 11 were in an immunocompromised state (5 with a history of chemotherapy for malignancy, 4 on immunosuppressive drugs or hormones, and 2 with confirmed HIV), 9 were in chronic respiratory failure or in prolonged stay in a medical care facility, and 3 were excluded due to lack of significant data. MV, mechanical ventilation; IMV, invasive mechanical ventilation; NIMV, noninvasive mechanical ventilation; ARDS, acute respiratory distress syndrome.

A total of 439 ICU patients were investigated during the study period, of whom 211 (48.06%) met the diagnostic criteria for sepsis or septic shock while in the ICU. Hypoxic respiratory failure was present in 116 (54.98%) of these patients according to the Berlin ARDS criteria, but 42 (36.21%) of them were not mechanically ventilated, and 74 (63.69%) were mechanically ventilated (both NIMV and IMV). Of these 74 patients, a total of 40 patients were eventually enrolled in the study (**Figure 1**). 25 (62.5%) and 15 (37.5%) patients in the study presented with mild to moderate and moderate to severe ARDS, respectively. None of the enrolled patients received extracorporeal membrane oxygenation (ECMO), and 4 patients were ventilated in the prone position. A total of 9 patients died at 28 days of follow-up, representing a mortality rate of 22.5%. All included patients were free of Coronavirus Disease 2019 (COVID-19).

### Characteristics and factors influencing the mortality of patients with sepsis-related ARDS

To analyze the factors associated with death in sepsis-related ARDS, the patients were divided into a survival group and a death group, and the characteristics of the patients were analyzed (**Table 1**). Forty patients with sepsis-related ARDS were included in this study. Baseline information was similar in both groups. The rank sum test showed that the number of patients with moderately severe ARDS was significantly higher within the death group (p=0.04); compared to the survival group, patients in the death group had higher white blood cell (WBC) counts (p=0.04). Among the cytokines, the levels of serum interleukin (IL)-4 (p=0.013), IL-6 (p=0.008), tumor necrosis factor (TNF)-α (p=0.031), interferon (IFN)-γ (p= 0.007) and IL-17A (p=0.014) were higher in the death group than in the survival group. Meanwhile, the CD16^bright^CD62L^dim^ neutrophil count was significantly higher in the death group (p=0.043) than in the survival group.

**Table 1:** Patient characteristics at baseline.

	Survivors (n=31)	Non-survivors (n=9)	Z/x^2^	p value
Patient characteristics				
Age years	62(46-69)	62(51-70.5)	-0.357	0.721
Males	18(58.06%)	7(77.78%)	1.157	0.282
BMI kg.m^-2^	25.4(23.18-28.5)	26.47(24.94-28.59)	-0.551	0.582
SOFA score	6(5-9)	10(5-12)	-1.259	0.208
APACHE II score	18(13-25)	24(15.5-29.5)	-1.298	0.194
GCS	11(7-15)	11(5.5-15)	-0.049	0.961
Application of vasoactive drugs, n (%)	12(38.71%)	7(77.78%)	–	0.06
Comorbidity no.				
Chronic respiratory disease	3(9.68%)	0	–	1
Diabetes mellitus	8(25.81%)	4(44.44%)	1.154	0.283
Chronic cardiovascular disease	11(35.48%)	3(33.33%)	0.014	0.905
Neurological disease	7(19.35%)	4(44.44%)	1.672	0.196
Chronic liver disease	2(6.45%)	1(11.11%)	–	0.545
Chronic kidney disease	2(6.45%)	0	–	1
Number of comorbidities, median(range)	1(0-2)	2(0-2.95)	-0.662	0.508
Cause of ICU admission				
Pneumonia	8(25.81%)	1(11.11%)	0.864	0.353
No-lung infection	10(32.26%)	3(33.33%)	0.004	0.952
Trauma	10(32.26%)	1(11.11%)	1.564	0.211
Pancreatitis	2(6.45%)	1(11.11%)	–	0.545
Other	1(3.23%)	3(33.33%)	–	0.03
Source of infection, n (%)				
Site of infection				
Pneumonia	21(67.74%)	7(77.78%)	0.335	0.563
Intra-abdominal infection	9(29.03%)	1(11.11%)	1.195	0.274
Urinary tract infection	3(9.68%)	0	–	1
Skin and soft tissue infection	3(9.68%)	1(11.11%)	–	1
Intestinal infection	0(0%)	1(11.11%)	–	0.225
Hemoculture-positive*	7(22.58%)	3(33.33%)	0.43	0.512
Laboratory findings				
Blood gases				
PaO_2_/FiO_2_ mmHg	220(140-280)	110(85-287.5)	-1.573	0.116
PaCO_2_ mmHg	36(27-43)	34(31-45.5)	-0.373	0.709
Lactate, mmol/L	1.3(0.9-1.7)	1.6(1-2.2)	-0.682	0.496
ARDS severity: Moderate to severe (PaO_2_/FiO_2<_150 mmHg) n (%)	9(29.03%)	6(66.67%)	4.215	0.04
Routine blood				
Neutrophil count (×10^9^/L)	7.31(5.8-12.4)	12.49(8.72-17.62)	-1.862	0.063
Lymphocyte count(×10^9^/L)	0.91(0.51-1.3)	1.09(0.625-1.58)	-0.940	0.347
NLR	11.89(7.83-18.21)	9.99(6.61-13.73)	-0.599	0.549
White blood cell count(×10^9^/L)	8.83(7.5-14)	14.3(10.13-20.08)	-2.057	0.04
Infection marker				
Procalcitonin (PCT) ng/mL	2.54(0.22-14.78)	7.4(4.21-27.23)	-1.927	0.054
(1,3)-beta-D-glucan test (%)	8(25.81%)	3(33.33%)	0.198	0.656
C-reactive protein mg/L	108.42 (54.81-158.55)	139.83 (118.02-351.47)	-1.862	0.063
Cytokines				
IL-2 pg/mL	2.13(1.52-2.62)	4.06(1.79-5.75)	-1.768	0.077
IL-4 pg/mL	2.32(1.84-3.85)	5.3(2.75-6.91)	-2.475	0.013
IL-6/Log2 pg/mL	5.64(4.14-7.59)	8.54(6.94-13.05)	-2.671	0.008
IL-8 pg/mL	10.86(3.26-30.29)	41.88(14.02-68.6)	-1.911	0.56
IL-10 pg/mL	10.38(3.72-56.53)	17.67(10.39-47.3)	-0.904	0.366
TNF-α pg/mL	1.84(1.37-2.81)	3.29(2.49-6.96)	-2.161	0.031
IFN-γ pg/mL	2.6(2.29-3.17)	4.45(3.3-8.12)	-2.721	0.007
IL-17A pg/mL	7.03(4.81-13.34)	15.32(7.88-25.28)	-2.455	0.014
Immune cells				
CD3^+^ T lymphocyte count/ul	543.5 (430.33-906.55)	378.9(274.1-850.5)	-0.850	0.395
CD4^+^ T lymphocyte count/ul	307.45 (214.4-444.43)	271.4(150.7-620.6)	-0.142	0.887
CD8^+^ T lymphocyte count/ul	201.6 (118.25-297.5)	124.8(96.6-229.9)	-0.945	0.345
CD4/CD8	1.99(0.93-2.69)	1.75(0.98-2.7)	-0.425	0.671
Treg (%)	7.59(5.27-11.3)	5.9(4.75-14.63)	-0.35	0.726
B lymphocyte count/ul	133.6(82.95-211.65)	92.85(67.28-227.75)	-0.875	0.382
NK cell count/ul	83.2(27.4-98.73)	52.1(23.33-81.5)	-0.852	0.394
CD16^bright^CD62L^dim^ PMN (%)	4.19(2.27-19.4)	15.5(4.79-26.5)	-1.733	0.083
CD16^bright^CD62L^dim^ PMN count (×10^9^/L)	0.54(0.16-1.74)	2.36(0.7-3.68)	-2.024	0.043

Data are expressed as n (%) or median (interquartile range) unless otherwise stated. *Hemoculture-positive includes unexplained bloodstream infections, and secondary bloodstream infections. BMI: Body mass index. SOFA score: Sequential Organ Failure Assessment. APACHE II score: Acute Physiology and Chronic Health Evaluation II score. GCS: Glasgow Coma Scale. PaO_2_: Arterial partial pressure of oxygen. PaCO_2_: Arterial blood carbon dioxide partial pressure. FiO2: inspiratory oxygen fraction. ARDS: Acute Respiratory Distress Syndrome. NLR: neutrophil to lymphocyte ratio. IL: interleukin. TNF: tumor necrosis factor. IFN: interferon. PMN: polymorphonuclear neutrophil.

Univariate analysis showed (**Table 2** and **Supplementary Table S2**, **Supplementary Figure S1**) that patients with sepsis-related ARDS who died within 28 days had higher SOFA scores at baseline, higher rates of vasoactive drug use, worse oxygenation indices, ARDS severity more frequently in the moderate to severe range, and higher WBC counts and neutrophil counts as well as higher levels of PCT, CRP, IL-2, IL-4, IL-6, IL-8, TNF-α, IFN-γ, and IL-17A. Patients with sepsis-related ARDS who had baseline proportions of the percentage of the CD16^bright^CD62L^dim^ neutrophil subtype to total neutrophils in peripheral blood (hereafter referred to as CD16^bright^CD62L^dim^ neutrophil subtype percentage) >3.73% and CD16^bright^CD62L^dim^ neutrophil counts >2.62×10^9^/L exhibited significantly increased 28-day mortality.

**Table 2:** Univariate analysis (P ≤ 0.05).

Subjects	Cutoff	N	Death No.(%)	Mean survival time	p value
**SOFA score**	≤8	26	3 (11.54%)	26.692	0.013
	>8	14	6 (42.86%)	19.571	
**Application of vasoactive drugs**	NO	21	2(9.52%%)	26.619	0.039
	YES	19	7(36.84%)	21.526	
**ARDS severity**	Moderate to severe	15	6 (40%)	20.0	0.021
	Mild to moderate	25	3 (12%)	26.720	
**PaO_2_/FiO_2_**	≤110	7	5 (71.43%)	14.286	0.000
	>110	33	4 (12.12%)	26.303	
**Neutrophil count (×10^9^/L)**	≤8.36	21	1 (4.76%)	27.095	0.006
	>8.36	19	8 (42.11%)	21	
**White blood cell count(×10^9^/L)**	≤9.3	20	1 (5%)	27.05	0.01
	>9.3	20	8 (40%)	21.35	
**Procalcitonin (PCT) ng/mL**	≤0.771	13	0	—	0.023
	>0.771	27	9 (33.33%)	—	
**C-reactive protein mg/L**	≤291	36	6 (16.67%)	25.472	0.000
	>291	4	3 (75%)	12.750	
**IL-2 pg/mL**	≤4.52	31	4 (12.9%)	26.258	0.000
	>4.52	4	4 (100%)	9.5	
**IL-4 pg/mL**	≤5.14	29	4 (13.79%)	25.931	0.002
	>5.14	6	4 (66.67%)	16.667	
**IL-6 pg/mL**	≤583	28	4 (14.29%)	25.857	0.007
	>583	7	4 (57.14%)	18.286	
**IL-8 pg/mL**	≤38.5	29	4(13.79%)	25.79	0.024
	>38.5	11	5(45.45%)	20	
**TNF-αpg/mL**	≤2.26	21	1 (4.76%)	27.667	0.001
	>2.26	14	7 (50%)	19.357	
**IFN-γpg/mL**	≤3.23	22	1 (4.55%)	27.182	0.001
	>3.23	13	7 (53.85%)	19.538	
**IL-17A pg/mL**	≤14.5	26	3 (11.54%)	26.385	0.004
	>14.5	9	5 (55.56%)	18.444	
**CD16^bright^CD62L^dim^ PMN (%)**	≤3.73	15	0	—	0.011
	>3.73	25	9 (36%)	—	
**CD16^bright^CD62L^dim^PMN count (×10^9^/L)**	≤2.62	29	4 (13.79%)	25.379	0.04
	>2.62	11	5 (45.45%)	21.091	

Data are expressed as n (%) or median (interquartile range) unless otherwise stated. SOFA score, Sequential Organ Failure Assessment; ARDS, Acute Respiratory Distress Syndrome; PaO_2_, Arterial partial pressure of oxygen; PaCO_2_, Arterial blood carbon dioxide partial pressure; FiO2, inspiratory oxygen fraction; IL, interleukin; TNF, tumor necrosis factor; IFN, interferon; PMN, polymorphonuclear neutrophil.

### Factors associated with infectious complications

Multifactorial COX regression analysis showed (**Table 3**) that baseline IL-8 (HR=3.852, 95% CI 1.561-9.508, p=0.003) and the percentage of the CD16^bright^CD62L^dim^ neutrophil subtype (HR=5.305, 95% CI 1.986-14.165, p=0.001) were independent risk factors for the development of infectious complications in patients with sepsis-related ARDS.

**Table 3:** Multifactorial COX regression analysis.

Subjects	B	Standard error	Ward	degrees of freedom	P	HR	95% CI
**IL-8**	1.349	0.461	8.560	1	0.003	3.852	1.561-9.508
**CD16^bright^CD62L^dim^ PMN (%)**	1.669	0.501	11.085	1	0.001	5.305	1.986-14.165

IL, interleukin; PMN, polymorphonuclear neutrophil.

**Figure 2:**
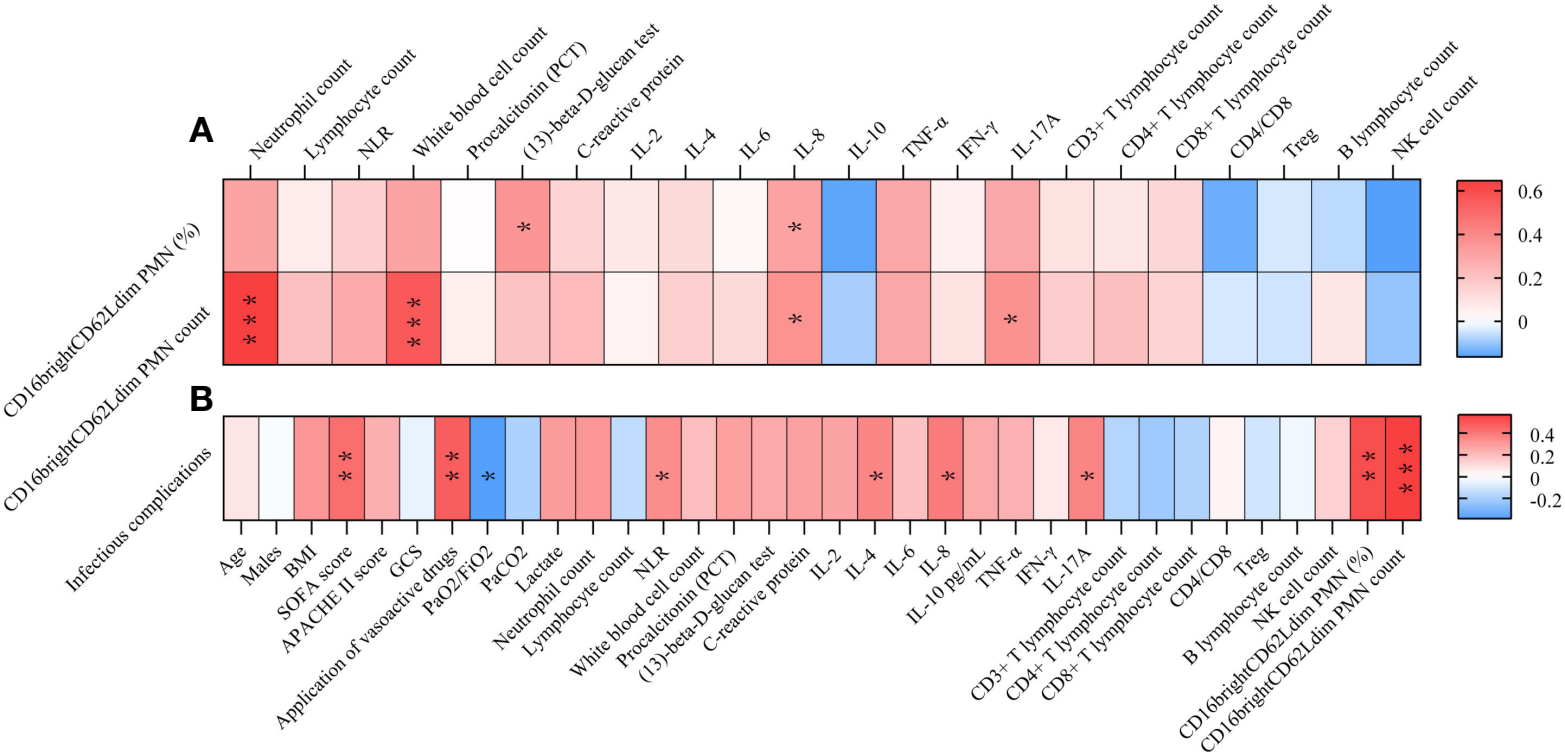
Correlation Analysis Heatmap. **(A)** Correlating factors of CD16^bright^CD62L^dim^ neutrophil subtypes. **(B)** Correlating factors of infectious complication. * P<0.05, **P<0.01. NLR, neutrophil to lymphocyte ratio; IL, interleukin; BMI, Body mass index; SOFA score, Sequential Organ Failure Assessment; APACHE II score, Acute Physiology and Chronic Health Evaluation II score; GCS, Glasgow Coma Scale; PaO_2_, Arterial partial pressure of oxygen; PaCO_2_, Arterial blood carbon dioxide partial pressure; FiO_2_, inspiratory oxygen fraction; TNF, tumor necrosis factor; IFN, interferon; PMN, polymorphonuclear neutrophil.

Correlation analysis showed (**Figure 2** and **Supplementary Table S4**) that the occurrence of infectious complications was associated with the use of vasoactive drugs (R=0.474, p=0.002), SOFA score (R=0.426, p=0. 006), neutrophil to lymphocyte ratio (NLR) (R=0.337, p=0.033), IL-4 (R=0.356, p=0.036), IL-8 (R=0.388, p=0.013), IL-17A (R=0.363, p=0.032), and the CD16^bright^CD62L^dim^ neutrophil percentage (R=0.521, p=0.001) and count (R=0.565, p=0.000) showed a positive correlation and a negative correlation with the oxygenation index (R=-0.391, p=0.013).

### Predictive ability of CD16^bright^CD62L^dim^ neutrophil subtypes for infection and death and Factors associated with CD16^bright^CD62L^dim^ neutrophil subtypes

**Figure 3:**
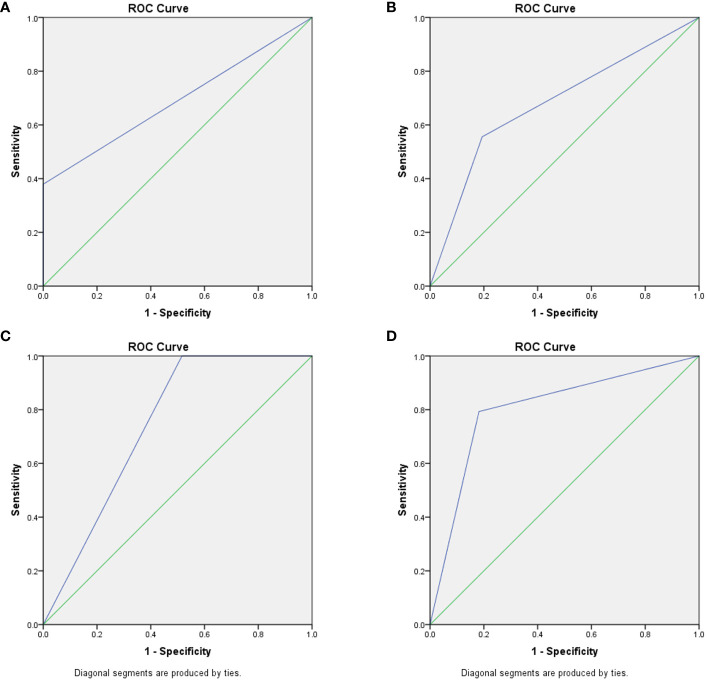
Predictive ability of CD16^bright^CD62L^dim^ neutrophil subtypes for infection and death. **(A)** CD16^bright^CD62L^dim^ neutrophil subtypes counts ROC curves predicting complications of infection, AUC of 0.69 (95% CI 0.526-0.853, P=0.067). **(B)** CD16^bright^CD62L^dim^ neutrophil subtypes counts ROC curves predicting death within 28 days, AUC of 0.681 (95% CI 0.468-0.894, P=0.102). **(C)** percentage of CD16^bright^CD62L^dim^ neutrophil subtypes ROC curves predicting complications of infection, AUC of 0.806 (95% CI 0.147-0.964, P=0.003). **(D)** percentage of CD16^bright^CD62L^dim^ neutrophil subtypes ROC curves predicting death within 28 days, AUC of 0.742 (95% CI 0.589-0.895, P=0.029).

In patients with sepsis-associated ARDS, the percentage of CD16^bright^CD62L^dim^ neutrophil subtypes predicted an AUC of 0.806 (95% CI 0.147-0.964, P=0.003) for the development of infectious complications, and 0.742 (95% CI 0.589-0.895, P=0.029) for the prediction of death within 28 days; whereas CD16^bright^CD62L^dim^ neutrophil subtype counts predicted an AUC of 0.69 (95% CI 0.526-0.853, P=0.067) for the development of infectious complications and 0.681 (95% CI 0.468-0.894, P=0.102) for the prediction of death within 28 days (**Figure 3**).

Correlation analysis showed (**Figure 2** and **Supplementary Table S5**) that the percentage of CD16^bright^CD62L^dim^ neutrophils was positively correlated with fungal (1,3)-beta-D-glucan and IL-8. And CD16^bright^CD62L^dim^ counts were positively correlated with neutrophil counts, white blood cell counts, IL-8 and IL-17A.

## Discussion

The main findings of this study are as follows. First, we demonstrated, for the first time, that elevated CD16^bright^CD62L^dim^ neutrophil subtypes were present in patients with sepsis-associated ARDS and were associated with infectious complications and poor prognosis in patients. Second, we found that baseline IL-8 and the percentage of the CD16^bright^CD62L^dim^ neutrophil subtype were independent risk factors for the development of infectious complications in patients with sepsis- associated ARDS.

**Figure 4:**
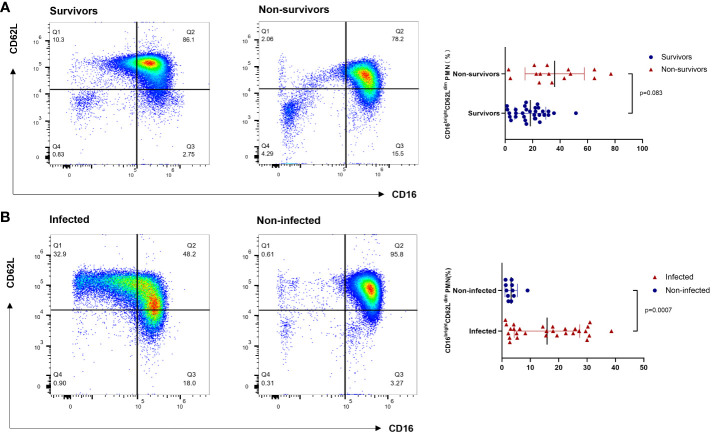
Flow cytometry results of CD16^bright^CD62L^dim^ neutrophil subtypes. **(A)** The survival and death groups. **(B)** The infectious complications and noninfectious complications groups. PMN: polymorphonuclear neutrophil.

Following a blow to the body (trauma or infection), a variety of pro- and anti-inflammatory cytokines are produced; measuring one or one class of inflammatory factors alone does not reflect the systemic immune status, but neutrophils respond to all of them as part of an eventual common pathway of systemic inflammation; this response can serve as a “simple” biomarker of complex systemic inflammation (16). CD16^bright^CD62L^dim^ neutrophils represent a recently identified neutrophil subtype associated with immunosuppressive functions. Contrary to conventional knowledge, CD16^bright^CD62L^dim^ are not senescent neutrophils; this population clusters separately in the proteomic profile and is a distinct class of neutrophil subpopulation, the origin of which is currently unknown (10). Previous studies have also found that CD16^bright^CD62L^dim^ neutrophils can inhibit T-cell proliferation through local release of hydrogen peroxide on the T-cell surface and through the expression of integrin macrophage differentiation antigen 1 (Mac-1) on the surface of neutrophils (11). This neutrophil subtype has been detected in human models of experimental endotoxemia and trauma and in the blood of COVID-19 patients (17). Similarly, elevated proportions of heterogeneous neutrophil subtypes have been found in the peripheral blood of ARDS patients (14). Our findings confirm that elevations in neutrophils of the CD16^bright^CD62L^dim^ subtype can be detected in the peripheral blood of patients with sepsis-related ARDS. In addition, this study showed that CD16^bright^CD62L^dim^ neutrophils in patients with sepsis-related ARDS were significantly elevated in the death group, whereas the elevation of this subtype was associated with infectious complications in the patients (**Figures 2**, **4**). In contrast to septic shock, this neutrophil subtype is a better predictor of infection complications and death in our study (**Figure 3** and **Supplementary Figure S2**). Therefore, we hypothesize that the CD16^bright^CD62L^dim^ neutrophil subtype shows more immunomodulatory features, which are important for maintaining the balance of the immune response (10, 11) and possibly even for tissue recovery (18). In our study, higher levels of baseline inflammatory markers were observed in the group of ARDS patients in the death group, which is consistent with previous studies (19). Therefore, given the immunomodulatory character of the CD16^bright^CD62L^dim^ neutrophil subtype, we hypothesize that its marked elevation in the ARDS death group may also play a role in modulating the more severe immune imbalance. However, the presence of many circulating CD16^bright^CD62L^dim^ neutrophils with immunosuppressive properties may also have another aspect. Clinical studies have found that CD16^bright^CD62L^dim^ neutrophils are associated with late-onset infections and the development of multiple organ dysfunction syndrome (MODS) in patients with severe trauma (12, 13). Our study also identified that CD16^bright^CD62L^dim^ neutrophil subtypes were associated with infectious complications in patients with ARDS (**Figure 2**). The presence of large neutrophil subpopulations with immunosuppressive properties plays an important role in the patient’s susceptibility status, leading to exacerbation of infection or the development of new opportunistic infections, contributing to further immune dysfunction and ultimately to the development of PICS and increased mortality.

PICS is often associated with recurrent infections and poor incisional healing and is associated with delayed hospitalization and high overall mortality (5). In this study, the percentage of CD16^bright^CD62L^dim^ subtypes to total neutrophils in peripheral blood neutrophils and IL-8 were found to be independent risk factors for the development of infectious complications in patients with sepsis-associated ARDS, and the combination of these two factors can be used to predict the development of infectious complications in patients with ARDS. CD16^bright^CD62L^dim^ neutrophil subtype exhibits mature neutrophils with typical multiple segmented nuclei. Their cell surface expression of the adhesion molecule CD62L (also known as L-selectin) is reduced, which decreases neutrophil drive toward sites of inflammation (20). In our study, the percentage of CD16^bright^CD62L^dim^ subtypes was found to correlate with infectious complications and could predict the occurrence of infectious complications, which reaffirms their important role in immune regulation in patients with ARDS. IL-8 is a pro-inflammatory chemokine that specifically chemotaxis neutrophils. However, in oncology studies, high levels of IL-8 in patients have been found to be associated with higher Myeloid-derived suppressor cells (MDSCs) infiltration, poorer T-cell function, and weaker antigen presentation.IL-8 has an inhibitory effect on adaptive immunity, affecting antigen presentation and effector T-cell activity, and tumor-derived IL-8 has also been associated with infiltration of polymorphonuclear neutrophil (PMN)- Myeloid-derived suppressor cells (MDSCs) (21). In this study we also found that elevated levels of IL-8 were associated with infectious complications in patients with sepsis-associated ARDS. The levels of IL-8 and CD16^bright^CD62L^dim^ neutrophil subtypes may provide us with a new reliable and easily measurable option for detecting immune levels in patients with sepsis-associated ARDS.

In this study, we investigated the factors influencing the development of infectious complications in patients with sepsis-associated ARDS. Our results showed that correlates promoting the development of infectious complications in patients with sepsis-associated ARDS included SOFA score, oxygenation index, the application of vasoactive drugs, NLR, IL-4, and IL-17A, in addition to CD16^bright^CD62L^dim^ neutrophils and IL-8 (**Figure 2** and **Supplementary Table S5**). Both the SOFA score and oxygenation index reflect the severity of the patient’s disease, so the findings may reflect the relationship between the severity of the disease and the patient’s infectious complications. Additionally, we found that infectious complications were associated with vasoactive drug application. In the treatment of sepsis, the preferred vasoactive drug is norepinephrine (NE). However, one should also be aware of the relationship between NE and immunosuppression. Numerous studies have confirmed that NE may be associated with immunosuppression in sepsis. NE exerts anti-inflammatory effects through β-adrenergic receptors (22), and *in vitro*, NE reduces lipopolysaccharide (LPS)-stimulated TNF-α and IL-6 production, increases the release of the anti-inflammatory factor IL-10 (23), and inhibits the cytotoxicity of natural killer cells (24). Clinical studies have also shown that “permissive hypotension” strategies that reduce the use of catecholamines improve 90-day mortality in elderly critically ill patients, whereas maintaining high levels of mean arterial pressure (MAP ≥70 mmHg) with high loading doses of vasoactive drugs increases mortality in elderly patients with infectious shock (25). Our study found that the use of vasoactive drugs was one of the factors associated with the development of infectious complications in patients with sepsis-related ARDS. There are two presumed reasons for this: first, the application of NE may be a sign of the severity of the patient’s condition, with the more severe condition causing greater immune dysfunction and predisposing the patient to infectious complications. Second, the application of NE led to a further exacerbation of immunosuppression in patients with sepsis-related ARDS. Immunosuppression is an important factor contributing to secondary infections and poor prognosis in patients with sepsis (4, 9, 26, 27), so a reduction in the use of NE may be warranted; however, this must be confirmed by more high-quality clinical studies as well as by finding alternatives to NE. The NLR is also known as the neutrophil-lymphocyte stress factor (28). Studies have shown that the NLR is strongly associated with the severity of sepsis in patients (29, 30). IL-4 is an anti-inflammatory cytokine, which has been shown to play a protective role in acute lung injury by promoting the resolution of inflammation (31). IL-17 is an important pro-inflammatory cytokine involved in the activation and recruitment of a variety of immune cells (32), and the current study confirms that it is involved in the progression of sepsis (33). Elevated IL-4 and IL-17A may respond to the patient’s overall level of inflammation in conjunction with an altered immune status.

We also found that CD16^bright^CD62L^dim^ neutrophil counts were positively correlated with neutrophil counts, leukocyte counts, IL-8, and IL-17A. IL-8 is a pro-inflammatory chemokine that specifically chemotaxes neutrophils, while IL-17A also increases neutrophil recruitment. The elevated CD16^bright^CD62L^dim^ neutrophil counts in this study were positively correlated with the levels of leukocytes, neutrophil counts, IL-8, and IL-17A, which we hypothesized to be since the alteration in CD16^bright^CD62L^dim^ neutrophil counts was more affected by the elevated plasma neutrophil counts. Also, our analysis showed that CD16^bright^CD62L^dim^ neutrophil percentage was a better predictor of infection complications and death in patients. This also suggests that it is the elevated CD16^bright^CD62L^dim^ percentage that is more sensitive to the response to a patient’s immune status than the change in CD16^bright^CD62L^dim^ count. Meanwhile, CD16^bright^CD62L^dim^ percentages were positively correlated with IL-8 and Fungal β-D-glucan (**Figure 2** and **Supplementary Table S5**). Previous study showed a relationship between tumor-derived IL-8 and PMN-MDSCs infiltration (21). And whether CD16^bright^CD62L^dim^ neutrophil subtypes correlate with PMN-MDSCs requires further validation in prospective studies. Fungal β-D-glucan is a fungal broad-spectrum circulating marker that suggests the presence of invasive fungal infectious diseases and is particularly applicable to patients with impaired immune function. The positive correlation between CD16^bright^CD62L^dim^ percentage and fungal β-D-glucan level further suggests that the CD16^bright^CD62L^dim^ percentage better responds to the patient’s immune function.

This study has some limitations. First, our study was a single-center study, which requires caution in generalizing the findings to other settings. Second, ARDS as defined in Berlin requires the use of positive end expiratory pressure (PEEP) ≥ 5 cmH_2_O, so only patients receiving MV were included in this study; this may have led to a greater tendency to include patients with more severe disease. Third, we only tested indicators within 48 hours of the diagnosis of sepsis-related ARDS, and it may be more meaningful to perform monitoring at multiple time points. Fourth, due to the patients’ severe disease and poor tolerance to bronchoscopy, we only performed tests for neutrophil subtypes and inflammatory factors in blood and did not perform tests related to alveolar lavage fluid. Fifth, the different pathogens of the ICU patients enrolled may have an impact on the study results, but due to the small sample size of this study and the fact that most of the patients were infected with multiple pathogens, it was not possible to differentiate the statistics; in subsequent research, the sample size could be expanded or multicenter studies could be conducted (**Supplementary Table S6**).

## Conclusions

In patients with sepsis-related ARDS, an elevated CD16^bright^CD62L^dim^ neutrophil subtype is present, and this subtype correlates with poor prognosis and infectious complications. This may indicate that the CD16^bright^CD62L^dim^ neutrophil subtype correlates with immunosuppression and disease severity in ARDS patients. The percentage of CD16^bright^CD62L^dim^ neutrophil subtypes may serve as a predictor of the development of infectious complications in patients with ARDS. Thus, the CD16^bright^CD62L^dim^ neutrophil subtype may be a target for the detection and regulation of immune function in patients with ARDS.

## Data availability statement

The original contributions presented in the study are included in the article/**Supplementary Material**. Further inquiries can be directed to the corresponding author.

## Ethics statement

The studies involving humans were approved by The ethics committee of the First Hospital of Jilin University. The studies were conducted in accordance with the local legislation and institutional requirements. The participants provided their written informed consent to participate in this study.

## Author contributions

JZ: Conceptualization, Writing – original draft. CG: Data curation, Formal analysis, Writing – original draft. ZZ: Investigation, Project administration, Writing – original draft. DL: Software, Writing – original draft. LQ: Validation, Visualization, Writing – original draft. QX: Investigation, Methodology, Writing – original draft. GW: Data curation, Software, Writing – review & editing. TJ: Methodology, Supervision, Writing – review & editing. FW: Supervision, Writing – review & editing.
