# Supplementary material for: New findings on CD16brightCD62Ldim neutrophil subtypes in sepsis-associated ARDS: an observational clinical study
Source: Front Immunol. 2024 Mar 28;15:1331050. doi: 10.3389/fimmu.2024.1331050 (PMC11007181; doi:10.3389/fimmu.2024.1331050)
Supplement: Supplementary file 1 [file DataSheet_1.pdf]

Supplemental materials

Table S1: Cutoff values. The cutoff values were calculated using jamovi for each laboratory index grouping, the survival curves under different index groupings were plotted using Kaplan-Meier. BMI: Body Mass Index. APACHE II score: Acute Physiology and Chronic Health Evaluation II score. SOFA score: Sequential Organ Failure Assessment score. GCS: Glasgow Coma Scale. PaO_2: Arterial partial pressure of oxygen. FiO2: inspiratory oxygen fraction. $PaCO_2$: Arterial blood carbon dioxide partial pressure. NLR: neutrophil to lymphocyte ratio. IL: interleukin. TNF: tumor necrosis factor. IFN: interferon. PMN: polymorphonuclear neutrophil.

Subjects	Cutoff values	Statistics
BMI $kg.m^{-2}$	25.6	1.76
Age years	40	1.12
APACHE II score	18	1.92
SOFA score	8	2.37
GCS score	3	1.4
Lactate, mmol/L	1.5	1.17
PaO_2/FiO_2	110	3.51
$PaCO_2$ mmHg	28	1.81
Neutrophil count ($\times10^9$/L)	8.36	2.74
Lymphocyte count ($\times10^9$/L)	0.55	1.47
NLR	14	1.76
White blood cell count ($\times10^9$/L)	9.3	2.57
Procalcitonin (PCT) ng/mL	0.771	2.33
C-reactive protein mg/L	291	2.77
IL-2 pg/mL	4.52	3.91
IL-4 pg/mL	5.14	2.76
IL-6 pg/mL	583	2.82
IL-8 pg/mL	38.5	2.23
IL-10 pg/mL	7.39	2
TNF-α pg/mL	2.26	3.15
IFN-γ pg/mL	3.23	3.26

IL-17A pg/mL	14.5	2.7
$CD3^+$ T lymphocyte count/ul	379	1.83
$CD4^+$ T lymphocyte count/ul	169	2.11
$CD8^+$ T lymphocyte count/ul	101	1.37
CD4/CD8	0.9	1.45
Treg (%)	6.39	1.28
B lymphocyte count/ul	97.6	1.67
$CD16^{bright}CD62L^{dim}$ PMN (%)	3.73	2.6
$CD16^{bright}CD62L^{dim}$ PMN count ($\times10^9$/L)	2.29	2.26

Table S2: Univariate analysis (P＞0.05). BMI: Body Mass Index. APACHE II score: Acute Physiology and Chronic Health Evaluation II score. GCS: Glasgow Coma Scale. ARDS：Acute Respiratory Distress Syndrome. PaCO2: Arterial blood carbon dioxide partial pressure. NLR: neutrophil to lymphocyte ratio. IL: interleukin. TNF: tumor necrosis factor. IFN: interferon. PMN: polymorphonuclear neutrophil.

Subjects	Cutoff	N	Death No.（%）	Mean survival time	p-value
Gender	Male	25	7 (28%)	22.960	0.277
	Female	15	2 (13.33%)	26.267	
Age years	⩽40	4	0		0.287
	＞40	36	9 (25%)		
BMI kg.m^{-2}	⩽25.6	19	2 (10.53%)	26.684	0.079
	＞25.6	21	7 (33.33%)	21.952	
APACHE II score	⩽18	20	2 (10%)	26.65	0.054
	＞18	20	7 (35%)	21.75	
GCS score	⩽3	4	2 (50%)	18.25	0.127
	＞3	36	7 (19.44%)	24.861	
Lactate, mmol/L	⩽1.5	25	4(16%)	24.96	0.24
	＞1.5	15	5(33.33%)	22.933	
Lactate >2 mmol/L	＜2	31	6(19.35%)	25	0.33
	⩾2	9	3(33.33%)	21.444	
Direct ARDS	YES	24	5 (20.83%)	24.5	0.748
	NO	16	4 (25%)	23.75	
Fungal (1-3)-β-D glucan	No-elevated	29	6 (20.69%)	24.310	0.69
	Elevated	11	3 (27.27%)	23.909	
Infection complications	YES	29	6 (20.69%)	25.069	0.545

	NO	11	3 (27.27%)	21.909	
Fungal infection	YES	17	5 (29.41%)	23.941	0.441
	NO	23	4 (17.39%)	24.391	
$PaCO_2$ mmHg	≤28	9	0		0.082
	>28	31	9 (29.03%)		
Lymphocyte count(×10^9/L)	≤0.55	12	1 (8.33%)	27.417	0.154
	>0.55	28	8 (28.57%)	22.821	
NLR	≤14	25	7 (28%)	22.6	0.239
	>14	15	2 (13.33%)	26.867	
IL-10 pg/mL	≤7.39	10	0		0.053
	>7.39	25	8 (32%)		
$CD3^+$ T lymphocyte count/ul	≤379	9	4 (44.44%)	20.556	0.058
	>379	22	3 (13.64%)	25.409	
$CD4^+$ T lymphocyte count/ul	≤169	4	2 (50%)	17.75	0.102
	>169	27	5 (18.52%)	24.926	
$CD8^+$ T lymphocyte count/ul	≤101	7	3 (42.86%)	21.857	0.164
	>101	24	4 (16.67%)	24.625	
CD4/CD8	≤0.9	6	0		0.165
	>0.9	25	7 (28%)		
Treg (%)	≤6.39	12	4 (33.33%)	21.667	0.195
	>6.39	15	2 (13.33%)	25.867	
B lymphocyte count/ul	≤97.6	10	4 (40%)	21.1	0.089
	>97.6	17	2 (11.76%)	25.706	

Table S3: Log-rank test for survival curves. The survival curves under different index groupings were plotted using Kaplan–Meier, the differences in survival curves were compared using the log-rank test. BMI: Body Mass Index. APACHE II score: Acute Physiology and Chronic Health Evaluation II score. SOFA score: Sequential Organ Failure Assessment score. PaO_2: Arterial partial pressure of oxygen. FIO2: inspiratory oxygen fraction. PaCO2: Arterial blood carbon dioxide partial pressure. WBC: white blood cell count. PCT: Procalcitonin. CRP: C-reactive protein. IL: interleukin. TNF: tumor necrosis factor. IFN: interferon. PMN: polymorphonuclear

neutrophil.

Subjects	chi-square test	Degrees of freedom	p-value
BMI	3.079	1	0.079
APACHE II score	3.708	1	0.054
SOFA score	6.202	1	0.013
PaO_2/FiO_2	18.334	1	0.000
$PaCO_2$ mmHg	3.026	1	0.082
Neutrophil count ($\times 10^9$/L)	7.650	1	0.006
White blood cell count($\times 10^9$/L)	6.657	1	0.010
Procalcitonin (PCT) ng/mL	5.144	1	0.023
C-reactive protein mg/L	12.896	1	0.000
ARDS severity	5.299	1	0.021
Application of vasoactive drugs	4.274	1	0.039
IL-2 pg/mL	27.962	1	0.000
IL-4 pg/mL	9.425	1	0.002
IL-6 pg/mL	7.253	1	0.007
IL-8 pg/mL	5.130	1	0.024
IL-10 pg/mL	3.760	1	0.053
TNF-α pg/mL	10.878	1	0.001
IFN-γ pg/mL	11.477	1	0.001
IL-17A pg/mL	8.525	1	0.004
$CD3^+$ T lymphocyte count/ul	3.592	1	0.058
B lymphocyte count/ul	2.887	1	0.089
$CD16^{bright}CD62L^{dim}$ PMN (%)	6.510	1	0.011
$CD16^{bright}CD62L^{dim}$ PMN count ($\times 10^9$/L)	4.226	1	0.040

Table S4. Correlation analysis of infectious complications.

Subjects	Infections	
	R	p value
Patient characteristics		
Age years	0.075	0.644
Males	-0.014	0.929
BMI $kg.m^{-2}$	0.308	0.053
SOFA score	0.426	0.006
APACHE II score	0.236	0.143
GCS	-0.052	0.751
Application of vasoactive drugs	0.474	0.002

Laboratory findings		
Blood gases		
PaO_2/FiO_2mmHg	-0.391	0.013
$PaCO_2$mmHg	-0.187	0.248
Lactate, mmol/L	0.292	0.068
Routine blood		
Neutrophil count	0.31	0.051
Lymphocyte count	-0.146	0.37
NLR	0.337	0.033
White blood cell count	0.192	0.236
Infection marker		
Procalcitonin (PCT)	0.279	0.081
(1,3)-beta-D-glucan test	0.254	0.114
C-reactive protein	0.276	0.084
Cytokine		
IL-2	0.272	0.114
IL-4	0.356	0.036
IL-6	0.182	0.365
IL-8	0.388	0.013
IL-10	0.252	0.204
TNF- α	0.227	0.255
IFN- γ	0.067	0.746
IL-17A	0.363	0.032
Immune cell		
$CD3^+$ T lymphocyte count	-0.175	0.347
$CD4^+$ T lymphocyte count	-0.222	0.229
$CD8^+$ T lymphocyte count	-0.183	0.325
CD4/CD8	0.032	0.865
Treg (%)	-0.109	0.59
B lymphocyte count	-0.033	0.872
NK cell count	0.134	0.514
$CD16^{bright}CD62L^{dim}$ PMN (%)	0.521	0.001
$CD16^{bright}CD62L^{dim}$ PMN count	0.565	0.000

ARDS: Acute Respiratory Distress Syndrome. BMI: Body Mass Index. SOFA score: Sequential Organ Failure Assessment. APACHE II score: Acute Physiology and Chronic Health Evaluation II score. GCS: Glasgow Coma Scale. PaO_2: Arterial partial pressure of oxygen. $PaCO_2$: Arterial blood carbon dioxide partial pressure. FiO2: inspiratory oxygen fraction. NLR: neutrophil to lymphocyte ratio. IL: interleukin.

TNF: tumour necrosis factor. IFN: interferon. PMN: polymorphonuclear neutrophil.

Table S5. Correlation analysis of $CD16^{bright}CD62L^{dim}$ neutrophils.

Subjects	$CD16^{bright}CD62L^{dim}$ (%)		$CD16^{bright}CD62L^{dim}$ count (×10^9/L)	
	R	p value	R	p value
Neutrophil count	0.311	0.051	0.646	0.0
Lymphocyte count	0.066	0.684	0.211	0.191
Neutrophil count/lymphocyte count	0.156	0.336	0.287	0.072
White blood cell count	0.307	0.054	0.559	0.0
Infection marker				
Procalcitonin (PCT)	0.01	0.951	0.056	0.732
(1,3)-beta-D-glucan test	0.356	0.024	0.201	0.213
C-reactive protein	0.147	0.364	0.232	0.149
Cytokine				
IL-2	0.079	0.654	0.042	0.811
IL-4	0.123	0.48	0.159	0.362
IL-6	0.025	0.888	0.122	0.486
IL-8	0.314	0.049	0.365	0.021
IL-10	-0.156	0.372	-0.085	0.626
TNF-α	0.292	0.089	0.295	0.086
IFN-γ	0.049	0.783	0.096	0.588
IL-17A	0.296	0.084	0.368	0.03
Immune cell				
$CD3^+$ T lymphocyte count	0.101	0.589	0.173	0.352
$CD4^+$ T lymphocyte count	0.084	0.654	0.216	0.243
$CD8^+$ T lymphocyte count	0.132	0.478	0.146	0.432
CD4/CD8	-0.141	0.449	-0.04	0.832
Treg (%)	-0.041	0.839	-0.047	0.816
B lymphocyte count	-0.065	0.748	0.083	0.681
NK cell count	-0.164	0.422	-0.102	0.619

NLR: neutrophil to lymphocyte ratio. IL: interleukin. TNF: tumour necrosis factor. IFN: interferon. PMN: polymorphonuclear neutrophil.

Table S6: Microbial etiology in the study population. Data are expressed as n or n (%).The percentage of pathogens in each group is related to the number of patients

with a pathogenic diagnosis. #: Polymicrobial cases: Klebsiella maltophilia + Klebsiella pneumoniae + Acinetobacter baumannii; Acinetobacter baumannii + Enterobacter cloacae + Candida albicans; Klebsiella pneumoniae + Aspergillus chimaerae; Citrobacter fowleri + Klebsiella pneumoniae + Acinetobacter baumannii; Escherichia coli + Acinetobacter baumannii + Acinetobacter calcoaceticus; Staphylococcus aureus + Candida albicans; Tropical Candida + Klebsiella pneumoniae. GNEB: gram-negative enterobacteria.

	Survivors	Non-survivors	Z/x^2	p-value
Subjects	31	9		
Patients with defined etiology	24(77.4%)	7(77.8%)	0.001	0.982
Streptococcus pneumoniae	1(4.2%)	0(0%)	-	1
Pseudomonas aeruginosa	1(4.2%)	1(14.3%)	-	0.406
Staphylococcus aureus	1(4.2%)	1(14.3%)	-	0.406
GNEB	8(33.3%)	2(28.6%)	-	1
Escherichia coli	3(12.5%)	1(14.3%)	-	1
Klebsiella pneumoniae	5(20.8%)	1(14.3%)	-	1
Acinetobacter	3(12.5%)	1(14.3%)	-	1
Fungi	2(8.3%)	0(0%)	-	1
Virus	1(4.2%)	0(0%)	-	1
Others	1(4.2%)	0(0%)	-	1
Polymicrobial cases#	6(25%)	2(28.6%)	0.036	0.849

Figure S1: Kaplan–Meier for survival curves. BMI: Body Mass Index. APACHE II score: Acute Physiology and Chronic Health Evaluation II score. SOFA score: Sequential Organ Failure Assessment score. PaO_2: Arterial partial pressure of oxygen. FIO2: inspiratory oxygen fraction. PaCO2: Arterial blood carbon dioxide partial pressure. WBC: white blood cell count. PCT: Procalcitonin. CRP: C-reactive protein. IL: interleukin. TNF: tumor necrosis factor. IFN: interferon. PMN: polymorphonuclear neutrophil.

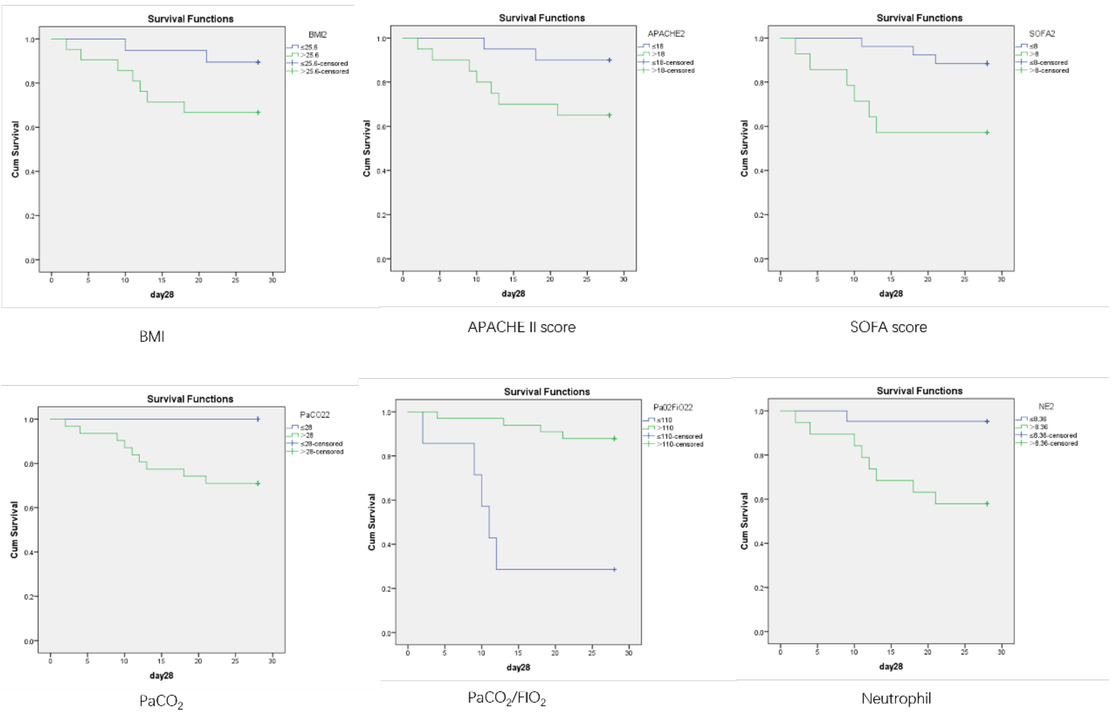

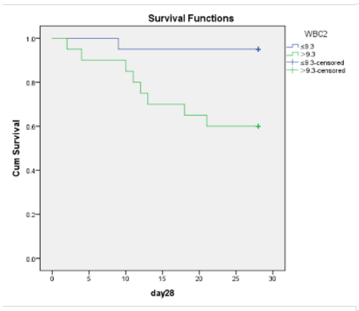

WBC

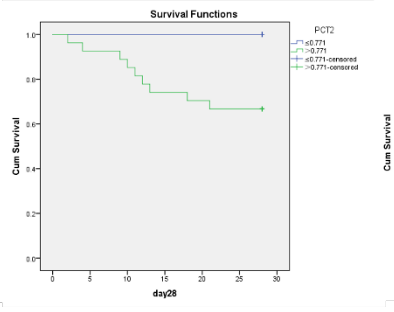

PCT

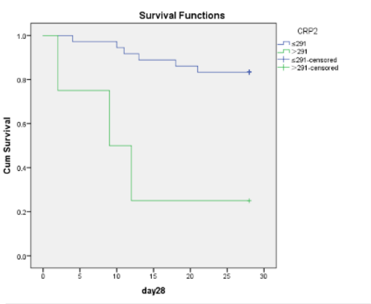

CRP

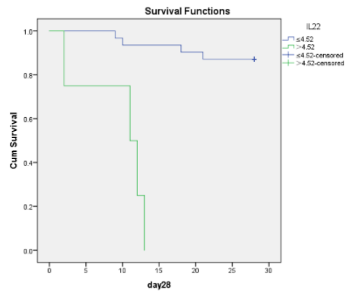

IL-2

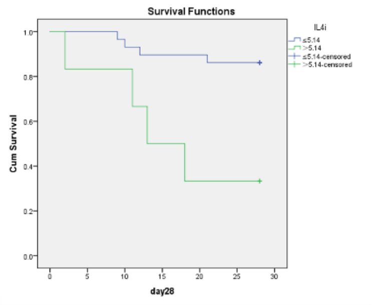

IL-4

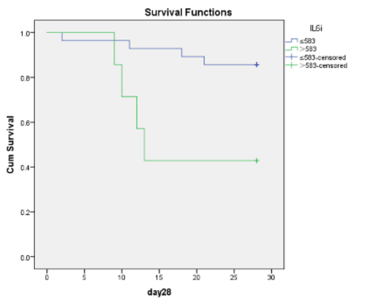

IL-6

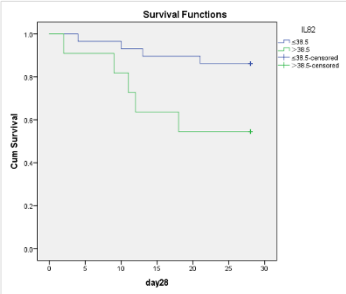

IL-8

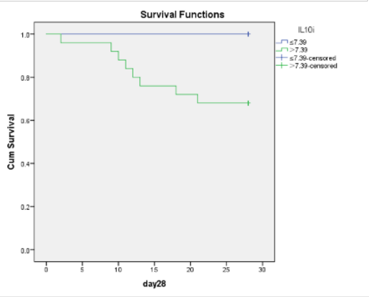

IL-10

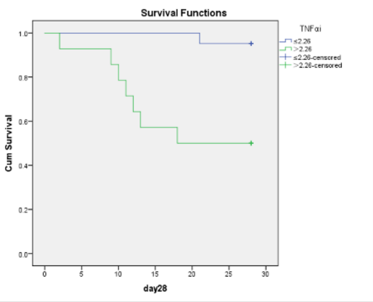

TNF-α

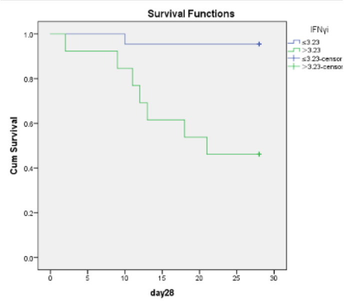

IFN-γ

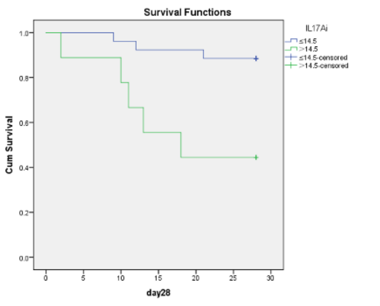

IL-17A

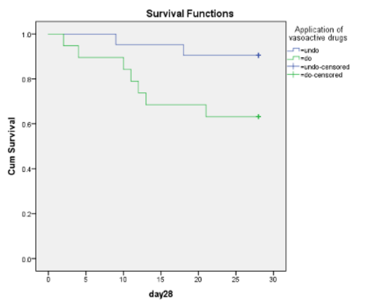

Application of vasoactive drugs

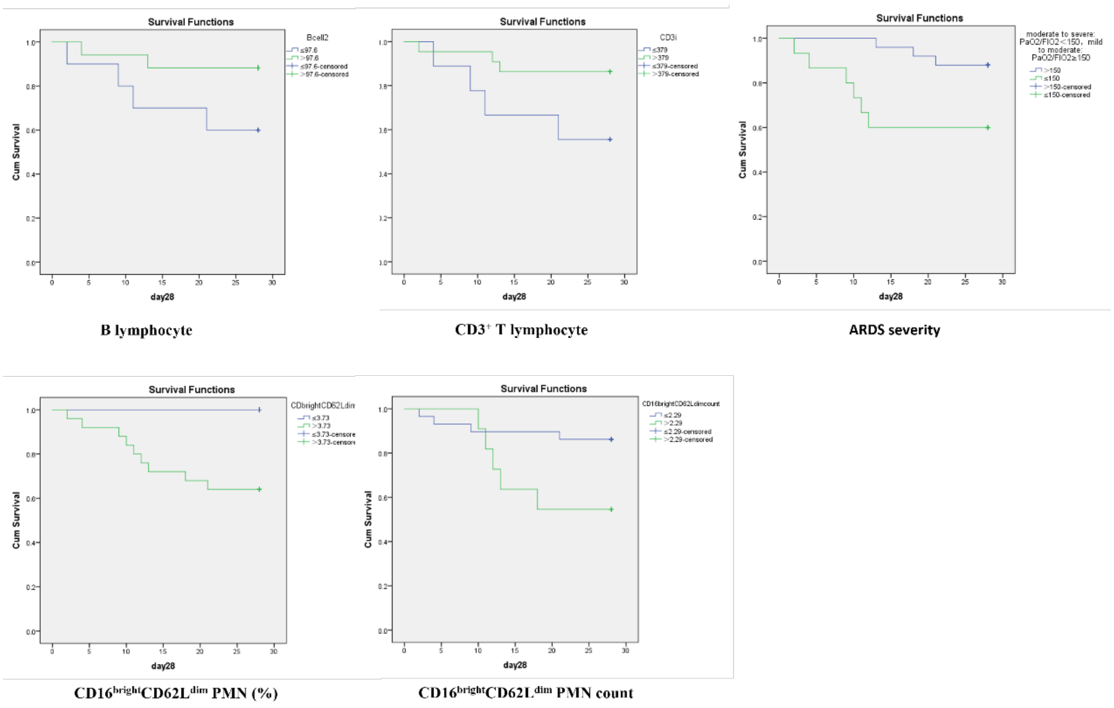

Figure S2. Predictive ability of CD16^{bright}CD62L^{dim} neutrophil subtypes for septic shock. A: The percentage of CD16^{bright}CD62L^{dim} neutrophil subtypes predicted an AUC of 0.644 (95% CI 0.47-0.818, P=0.119) of septic shock. B: CD16^{bright}CD62L^{dim} neutrophil subtypes count predicted an AUC of 0.612 (95% CI 0.436-0.787, P=0.228) for septic shock.

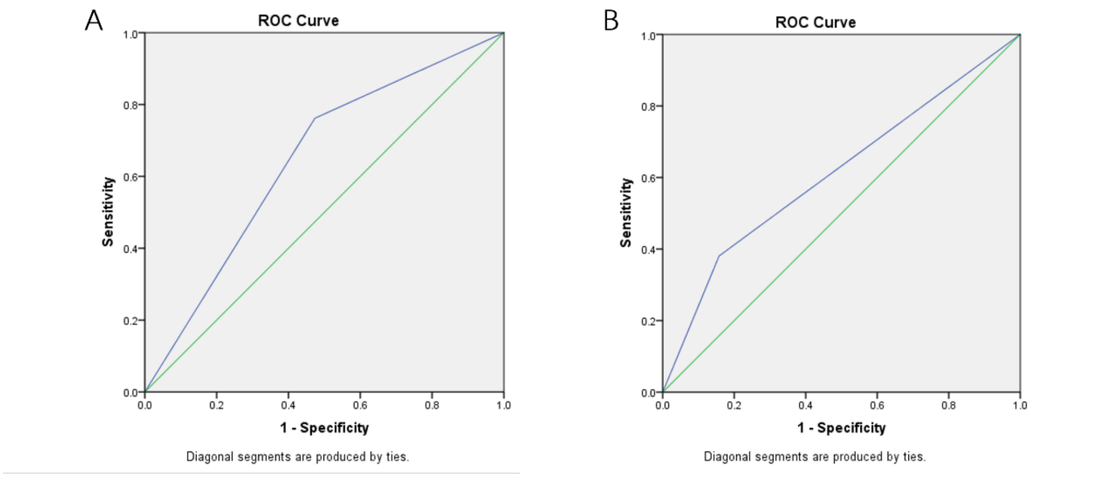

Definitions

ARDS and severity classification: according to the Berlin definition [1], ARDS is defined as new or worsening respiratory symptoms accompanied by bilateral pulmonary radiographic opacities that cannot be fully explained by fluid accumulation, lobber's/pulmonary atrophy or nodules that cannot be fully explained by heart failure or fluid overload, arterial partial pressure of oxygen (PaO_2)/inspiratory oxygen fraction (FiO2) ratio ≤300 mmHg, and positive end expiratory pressure (PEEP) or positive continuous airway pressure >5 cmH_2O. In this study, ARDS severity was classified as mild to moderate (150 mmHg < PaO_2/FiO_2 ≤300 mmHg) and moderate to severe (PaO_2/FiO_2 ≤150 mmHg) [2]. ARDS severity grouping (ratio of PaO_2/FiO_2) was noted; if the difference between day 1 and day 2 was large, the results of day 2 were used [3].

Sepsis: according to the SEPSIS-3 definition [4], sepsis is defined as life-threatening organ dysfunction caused by a dysregulated host response to infection. Organ dysfunction can be indicated by an increase of 2 or more points in the continuous (sepsis-related) organ failure assessment (SOFA) score. Septic shock is defined as existing in a patient who requires an elevating agent to maintain a mean arterial pressure of 65 mmHg or higher in the absence of hypovolemia, who has a serum lactate level greater than 2 mmol/L (> 18 mg/dL) and who is clinically determined to be in infectious shock.

Complications of infection: complications of infection were defined as those found after 5 days of enrolment that involve emergence of a new site of infection or

progression of a pre-existing infection to worsen or even life-threatening, including urinary tract infections, surgical site infections, fracture-related infections, skin infections, soft tissue infections, pneumonia, primary peritonitis, secondary peritonitis, intra-abdominal abscesses, unexplained bloodstream infections, and secondary bloodstream infections. All infections diagnosed by clinicians, whether radiological, clinical or microbiological, were documented in this study.

Immune abnormalities: patients receiving steroids or other immunosuppressive drugs for a prolonged period (> 3 months) or at high doses (> 0.5 mg/kg/day), solid organ transplant recipients, patients with solid tumours requiring chemotherapy in the last 5 years, patients with haematologic malignancies (regardless of the time since diagnosis and treatment), and patients with primary immunodeficiency [5].

References

1. Matthay MA, Zemans RL, Zimmerman GA, et al. Acute respiratory distress syndrome. Nat Rev Dis Primers 2019: 5: 18.
2. Bos LD, Cremer OL, Ong DS, et al. External validation confirms the legitimacy of a new clinical classification of ARDS for predicting outcome. Intensive Care Med 2015: 41: 2004-2005.
3. Gattinoni L, Pesenti A, Matthay M. Understanding blood gas analysis. Intensive Care Med 2018: 44: 91-93.
4. Singer M, Deutschman CS, Seymour CW, et al. The third international consensus definitions for sepsis and septic shock (Sepsis-3). JAMA 2016: 315: 801-810.

5. Azoulay E, Russell L, Van de Louw A, et al. Diagnosis of severe respiratory infections in immunocompromised patients. Intensive Care Med 2020: 46: 298-314.
